# 2,2′,5,5′-Tetra­chloro­benzidine

**DOI:** 10.1107/S1600536810030886

**Published:** 2010-08-11

**Authors:** Onome Ugono, Marcel Douglas Jr, Nigam P. Rath, Alicia M. Beatty

**Affiliations:** a Department of Chemistry and Biochemistry, University of Missouri–St Louis, St Louis, Missouri, USA

## Abstract

In the crystal structure of the title compound, C_12_H_8_Cl_4_N_2_, mol­ecules lie on crystallographic twofold axes at the centre of the C—C bonds linking the benzene rings, such that the asymmetric unit consists of a half-mol­ecule. The individual mol­ecules participate in inter­molecular N—H⋯N, N—H⋯Cl, C—H⋯Cl and Cl⋯Cl [3.4503 (3) Å] inter­actions.

## Related literature

For studies involving the use of benzidines in organic syntheses, see: Schwenecke & Mayer (2005 ▶). For studies on 2,2′,5,5′-tetra­chloro­benzidine in crystal engineering, see: Dobrzycki & Wozniak (2007 ▶, 2008 ▶). For our studies on related structures, see: Beatty *et al.* (2002*a* ▶, 2002*b* ▶); Ugono *et al.* (2009 ▶).
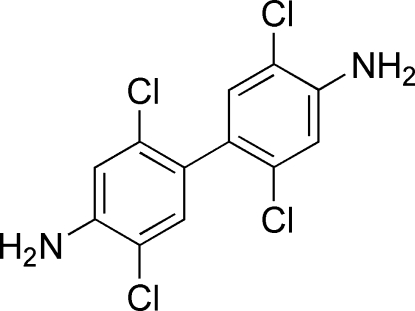


## Experimental

### 

#### Crystal data

C_12_H_8_Cl_4_N_2_
*M* *_r_* = 322.00
Monoclinic, 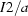

*a* = 17.2346 (11) Å
*b* = 3.8767 (2) Å
*c* = 18.1573 (19) Å
β = 94.872 (3)°
*V* = 1208.77 (16) Å^3^
*Z* = 4
Mo *K*α radiation
μ = 0.96 mm^−1^
*T* = 100 K
0.23 × 0.22 × 0.14 mm

#### Data collection

Bruker APEXII CCD diffractometer
Absorption correction: multi-scan (*SADABS*; Sheldrick, 1996 ▶) *T*_min_ = 0.806, *T*_max_ = 0.879
11878 measured reflections
2961 independent reflections
2595 reflections with *I* > 2σ(*I*)
*R*_int_ = 0.025

#### Refinement

*R*[*F* ^2^ > 2σ(*F* ^2^)] = 0.025
*wR*(*F* ^2^) = 0.075
*S* = 1.07
2961 reflections
82 parameters
H-atom parameters constrained
Δρ_max_ = 0.62 e Å^−3^
Δρ_min_ = −0.67 e Å^−3^

### 

Data collection: *APEX2* (Bruker, 2007 ▶); cell refinement: *SAINT* (Bruker, 2007 ▶); data reduction: *SAINT*; program(s) used to solve structure: *SHELXS97* (Sheldrick, 2008 ▶); program(s) used to refine structure: *SHELXL97* (Sheldrick, 2008 ▶); molecular graphics: *SHELXTL* (Sheldrick, 2008 ▶); software used to prepare material for publication: *SHELXTL* and *PLATON* (Spek, 2009 ▶).

## Supplementary Material

Crystal structure: contains datablocks global, I. DOI: 10.1107/S1600536810030886/fj2305sup1.cif

Structure factors: contains datablocks I. DOI: 10.1107/S1600536810030886/fj2305Isup2.hkl

Additional supplementary materials:  crystallographic information; 3D view; checkCIF report

## Figures and Tables

**Table 1:** Hydrogen-bond geometry (Å, °)

*D*—H⋯*A*	*D*—H	H⋯*A*	*D*⋯*A*	*D*—H⋯*A*
N1—H1*A*⋯Cl2^i^	0.88	2.79	3.3650 (8)	124
C3—H3⋯Cl1^ii^	0.95	2.71	3.5013 (9)	141
N1—H1*B*⋯N1^i^	0.88	2.90	3.2159 (12)	103

Symmetry codes: (i) 
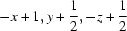
; (ii) 
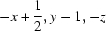
.

**Fig. 1.**
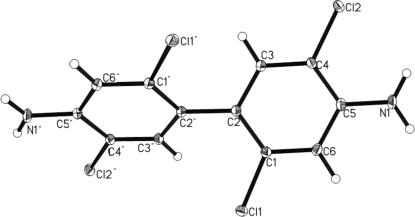
Molecular structure showing 50% probability displacement ellipsoid.

## supplementary crystallographic information

### Comment

Benzidines are of great importance in the pigments industry, as their derivatives are employed in the syntheses of a variety of azo-dyes (Schwenecke and Mayer 2005). Furthermore, the presence of two amine functionalities renders this class of molecules attractive to crystal engineers, who may wish to incorporate this class of rigid linear molecule in larger extended networks *via* hydrogen bonds (Dobrzycki and Wozniak 2008 a, b). During the course of experiments aimed at reacting the title compound with pyrazole-3,5-dicarboxylic acid, a crystalline phase was obtained and shown to be composed solely of 2,2',5,5'-tetrachlorobenzidine. A search of the Cambridge crystallographic structural database showed that this phase has not previously been reported. This molecule packs in monoclinic *I*2/a, a non-standard setting of *C*2/*c*, with one half of the molecule in the asymmetric unit. Very weak hydrogen bonding interactions exist in the structure; the increased lengths are probably due to the bulky chlorine atoms *ortho* to the amine functionalities.

### Experimental

A 20 ml scintillation vial was charged with 52.0 mg (0.30 mmol) of 3,5-pyrazole dicarboxylic acid monohydrate, which was dissolved in 5.0 ml of a 3:2 MeOH:H_2_O mixture affording a homogenous solution. To this solution was then added 48.3 mg (0.15 mmol) of 2,2',5,5'-tetrachlorobenzidine. The mixture obtained was filtered and the filtrate was allowed to slowly evaporate to yield colorless single crystals of 2,2',5,5'-tetrachlorobenzidine after a week.

### Refinement

All non hydrogen atoms were refined anisotropically. Phenyl hydrogen atoms were placed in calculated positions and treated with a riding model C–H= 0.95 Å, *U*_iso_(H_aryl_)= 1.2*U*_eq_(C) for aromatic carbons. The amine hydrogen atoms were also placed in calculated positions and refined using the riding model N–H= 0.88 Å, *U*_iso_(H_amine_)= 1.2*U*_eq_(N).

### Crystal data

**Table:**

C_12_H_8_Cl_4_N_2_	*F*(000) = 648
*M**_r_* = 322.00	*D*_x_ = 1.769 Mg m^−^^3^
Monoclinic, *I*2/*a*	Melting point = 309–311 K
Hall symbol: -I 2ya	Mo *K*α radiation, λ = 0.71073 Å
*a* = 17.2346 (11) Å	Cell parameters from 5249 reflections
*b* = 3.8767 (2) Å	θ = 4.5–36.4°
*c* = 18.1573 (19) Å	µ = 0.96 mm^−^^1^
β = 94.872 (3)°	*T* = 100 K
*V* = 1208.77 (16) Å^3^	Blocks, colorless
*Z* = 4	0.23 × 0.22 × 0.14 mm

### Data collection

**Table:**

Bruker APEXII CCD diffractometer	2961 independent reflections
Radiation source: fine-focus sealed tube	2595 reflections with *I* > 2σ(*I*)
graphite	*R*_int_ = 0.025
φ and ω scans	θ_max_ = 36.4°, θ_min_ = 3.1°
Absorption correction: multi-scan (*SADABS*; Sheldrick, 1996)	*h* = −28→28
*T*_min_ = 0.806, *T*_max_ = 0.879	*k* = −3→6
11878 measured reflections	*l* = −30→26

### Refinement

**Table:**

Refinement on *F*^2^	0 restraints
Least-squares matrix: full	H-atom parameters constrained
*R*[*F*^2^ > 2σ(*F*^2^)] = 0.025	*w* = 1/[σ^2^(*F*_o_^2^) + (0.0404*P*)^2^ + 0.5838*P*] where *P* = (*F*_o_^2^ + 2*F*_c_^2^)/3
*wR*(*F*^2^) = 0.075	(Δ/σ)_max_ < 0.001
*S* = 1.07	Δρ_max_ = 0.62 e Å^−^^3^
2961 reflections	Δρ_min_ = −0.67 e Å^−^^3^
82 parameters	

### Special details

**Table:**

Experimental. All H atoms were added in their calculated positions and were treated using appropriate riding models.
Geometry. All e.s.d.'s (except the e.s.d. in the dihedral angle between two l.s. planes) are estimated using the full covariance matrix. The cell e.s.d.'s are taken into account individually in the estimation of e.s.d.'s in distances, angles and torsion angles; correlations between e.s.d.'s in cell parameters are only used when they are defined by crystal symmetry. An approximate (isotropic) treatment of cell e.s.d.'s is used for estimating e.s.d.'s involving l.s. planes.
Refinement. Refinement of *F*^2^ against ALL reflections. The weighted *R*-factor *wR* and goodness of fit *S* are based on *F*^2^, conventional *R*-factors *R* are based on *F*, with *F* set to zero for negative *F*^2^. The threshold expression of *F*^2^ > σ(*F*^2^) is used only for calculating *R*-factors(gt) *etc*. and is not relevant to the choice of reflections for refinement. *R*-factors based on *F*^2^ are statistically about twice as large as those based on *F*, and *R*- factors based on ALL data will be even larger.

### Fractional atomic coordinates and isotropic or equivalent isotropic displacement parameters (Å^2^)

**Table:**

	*x*	*y*	*z*	*U*_iso_*/*U*_eq_	
Cl1	0.160889 (12)	0.57866 (5)	0.112011 (11)	0.01179 (5)	
Cl2	0.490222 (11)	−0.04833 (5)	0.098542 (11)	0.01279 (6)	
N1	0.42631 (4)	0.2547 (2)	0.23242 (4)	0.01399 (13)	
H1A	0.4109	0.3405	0.2736	0.017*	
H1B	0.4733	0.1666	0.2320	0.017*	
C1	0.25315 (5)	0.4094 (2)	0.10312 (5)	0.00977 (13)	
C2	0.27558 (5)	0.2934 (2)	0.03511 (4)	0.00940 (12)	
C3	0.35064 (5)	0.1556 (2)	0.03688 (4)	0.01012 (13)	
H3	0.3688	0.0715	−0.0077	0.012*	
C4	0.39925 (5)	0.1373 (2)	0.10113 (5)	0.00990 (13)	
C5	0.37670 (5)	0.2584 (2)	0.16844 (4)	0.01036 (13)	
C6	0.30195 (5)	0.3964 (2)	0.16774 (5)	0.01065 (13)	
H6	0.2842	0.4830	0.2123	0.013*	

### Atomic displacement parameters (Å^2^)

**Table:**

	*U*^11^	*U*^22^	*U*^33^	*U*^12^	*U*^13^	*U*^23^
Cl1	0.00978 (9)	0.01507 (9)	0.01067 (9)	0.00235 (6)	0.00183 (6)	0.00083 (6)
Cl2	0.00832 (9)	0.01894 (10)	0.01088 (10)	0.00207 (6)	−0.00065 (6)	0.00105 (6)
N1	0.0119 (3)	0.0218 (3)	0.0076 (3)	0.0000 (3)	−0.0027 (2)	−0.0006 (2)
C1	0.0089 (3)	0.0118 (3)	0.0086 (3)	0.0002 (2)	0.0006 (2)	0.0005 (2)
C2	0.0084 (3)	0.0118 (3)	0.0079 (3)	−0.0002 (2)	0.0000 (2)	0.0000 (2)
C3	0.0093 (3)	0.0127 (3)	0.0081 (3)	0.0001 (2)	−0.0002 (2)	−0.0006 (2)
C4	0.0076 (3)	0.0130 (3)	0.0088 (3)	0.0001 (2)	−0.0004 (2)	0.0003 (2)
C5	0.0098 (3)	0.0128 (3)	0.0082 (3)	−0.0021 (2)	−0.0009 (2)	0.0005 (2)
C6	0.0110 (3)	0.0135 (3)	0.0074 (3)	−0.0007 (2)	0.0005 (2)	−0.0005 (2)

### Geometric parameters (Å, °)

**Table:**

Cl1—C1	1.7402 (8)	C2—C3	1.3974 (11)
Cl2—C4	1.7295 (8)	C2—C2^i^	1.4872 (16)
N1—C5	1.3827 (11)	C3—C4	1.3791 (11)
N1—H1A	0.8800	C3—H3	0.9500
N1—H1B	0.8800	C4—C5	1.3948 (12)
C1—C6	1.3854 (12)	C5—C6	1.3940 (12)
C1—C2	1.3993 (12)	C6—H6	0.9500
			
C5—N1—H1A	120.0	C2—C3—H3	118.9
C5—N1—H1B	120.0	C3—C4—C5	121.97 (7)
H1A—N1—H1B	120.0	C3—C4—Cl2	119.03 (6)
C6—C1—C2	122.84 (8)	C5—C4—Cl2	118.98 (6)
C6—C1—Cl1	115.40 (6)	N1—C5—C6	121.10 (8)
C2—C1—Cl1	121.75 (6)	N1—C5—C4	122.30 (8)
C3—C2—C1	115.29 (7)	C6—C5—C4	116.56 (7)
C3—C2—C2^i^	120.00 (8)	C1—C6—C5	121.05 (8)
C1—C2—C2^i^	124.63 (8)	C1—C6—H6	119.5
C4—C3—C2	122.26 (8)	C5—C6—H6	119.5
C4—C3—H3	118.9		
			
C6—C1—C2—C3	1.26 (12)	C3—C4—C5—N1	−177.11 (8)
Cl1—C1—C2—C3	−178.33 (6)	Cl2—C4—C5—N1	4.10 (11)
C6—C1—C2—C2^i^	178.20 (6)	C3—C4—C5—C6	0.59 (12)
Cl1—C1—C2—C2^i^	−1.39 (9)	Cl2—C4—C5—C6	−178.20 (6)
C1—C2—C3—C4	−0.36 (12)	C2—C1—C6—C5	−1.26 (12)
C2^i^—C2—C3—C4	−177.46 (6)	Cl1—C1—C6—C5	178.35 (6)
C2—C3—C4—C5	−0.56 (13)	N1—C5—C6—C1	178.02 (8)
C2—C3—C4—Cl2	178.23 (6)	C4—C5—C6—C1	0.29 (12)

Symmetry codes: (i) −*x*+1/2, *y*, −*z*.

### Hydrogen-bond geometry (Å, °)

**Table:**

*D*—H···*A*	*D*—H	H···*A*	*D*···*A*	*D*—H···*A*
N1—H1A···Cl2^ii^	0.88	2.79	3.3650 (8)	124
C3—H3···Cl1^iii^	0.95	2.71	3.5013 (9)	141
N1—H1B···N1^ii^	0.88	2.90	3.2159 (12)	103

Symmetry codes: (ii) −*x*+1, *y*+1/2, −*z*+1/2; (iii) −*x*+1/2, *y*−1, −*z*.

### Table 2 Cl···Cl interactions (Å)

**Table:**

Cl1···Cl2^i^	3.4503 (3)

Symmetry code: (i) -1/2+x, 1-y, z.
